# Review of Cost-Effectiveness Analysis of Medical Treatment For Myocardial Infarction

**Published:** 2011

**Authors:** Yanmei Liu, Koustuv Dalal

**Affiliations:** 1 MPH Student, Karolinska Institutet, Sweden; 2 Centre for Medical Technology Assessment, Linkoping University, Linkoping, Sweden

**Keywords:** Cost effectiveness Analysis, Heart, Medicine

## Abstract

**Objectives:**

Myocardial infarction (MI) is a leading cause of death in both the industrialized and developing countries globally. The economic evaluation of MI is undertaken to rationale the allocation of scarce healthcare resource. The objective is to review cost-effectiveness analysis of MI with medications.

**Methods:**

We searched PubMed using the key words: “cost effectiveness analysis”and “myocardial infarction” After applying the selection criteria, eight articles were selected for the present study.

**Results:**

Out of eight articles, five had studied thrombolytic agents. All of these papers clearly explain the costs and benefits of different drugs for MI. ICER was assessed in six out of the eight articles to compare the costs and health effects between alternative medications. ICER was expressed in different effect units.

**Conclusions:**

This study found that various medications including thrombolytic agents, ACEI and heparin are administered to treat MI in many countries. It is also found that five of eight studies focus on thrombolytic therapies. It implies that thrombolytic is generally very cost effective for MI to the whole society.

## INTRODUCTION

Nowadays, myocardial infarction (MI) is one of the major health problems in terms of morbidity and mortality in both developed and developing world.^1^ On basis of the redefined definition^2^ in 2000, MI is the irreversible necrosis of heart muscle secondary to prolonged ischemia. There are a number of medications and surgical interventions adopted to restore the normal coronary blood flow for patients with MI in the early and post MI treatment. Furthermore, the beneficial efficacy of these therapies has been being observed in different trials in various settings. For instance, the use of aspirin has been shown to reduce Mortality from MI for years,^3^ despite the fact that some patients are allergic or resistant to the aspirin; thrombolytic agents such as streptokinase, anistreplase or Anisoylated plasminogen streptokinase activator complex (APSAC), and alteplase (rt-PA) have been proved to be cost-effective in many studies^4^–^7^ since the 1980s; the efficacy of early Lisinopril (angiotensin- converting enzyme inhibitor, ACEI) use in patients with acute myocardial infarction (AMI)^8^ was assessed in the GISSI-3 Trail; in the ExTRACT-TIMI25 Trial^9^ –^10^ implemented in 48 countries, enoxa-parin was found to have better clinical benefits than unfractionated heparin (UFH) as adjunctive therapy for fibrinolysis in patients with ST-segment elevation myocardial infarction (STEMI. Other important therapies, including beta blockers,^3^,^11^ nitrates, percutaneous coronary intervention (PCI)^3^,^11^ and Coronary artery bypass grafting (CABG),^3^ etc. have been proved to have positive effect on the reduction of the mortality rate in MI.

It is known to all, the costs of healthcare/medical interventions are rising enormously worldwide, regardless of the development of modern medical technology. Economic evaluationshave been emerged as important tool to allocate the scarce resources efficiently and rationally.^12^–^13^ Methods of economic evaluations including cost-effectiveness, cost-utility, and cost-benefit analyses are applied to assess health care programs in many arenas containing treatments of MI which impose a high burden on economics worldwide.^14^ In relative to various therapies of MI, it is important to measure and compare the different costs, health outcomes and efficacy, then identify the most cost-effective treatment which enables the decision makers to opt from a range of alternatives. It also implies efficient use of health care resources. Like other important health field, MI has also produced economic evaluations in the scientific articles. The aim of this study is to perform a review of the cost-effectiveness analysis of treatments of MI with medications.

## METHODS

We searched PubMed with the following search terms: cost effectiveness analysis + myocardial infarction. We found 1099 articles. By limiting the searching terms for randomized clinical trail (RCT), humans and English language we found 133 articles for further review according to the following inclusion and exclusion criteria.

### 

#### Inclusion criteria

All the selected studies should cover both costs and benefits of drug treatments for MI. Firstly, by reviewing the titles of articles, only those containing terms of “cost-effectiveness” “medications” “myocardial infarction” were retained. Next, studies on the cost-effectiveness of certain pharmaceuticals to treat myocardial infarction were included for the full text review. Finally, if costs and outcome were indicated clearly in the articles, they were included.

#### Exclusion criteria

Reference studies were excluded if patients of other coronary artery diseases participated in the studies. Studies were excluded if: they were of post-infarction or preventive strategies; they compared medication treatment with surgical treatment.

According to the definition of cost-effectiveness analysis, it’s not a full economic evaluation when only costs or effects were studied.^14^ Therefore we have excluded those studies with only cost or effect.

Reference studies were excluded if found not to be about economic evaluations or cost-effectiveness analysis. And studies did not have sufficient details of economic evaluations were excluded as well.

#### Final selection of the articles

With those criteria we have selected 13 articles after scrutinizing the titles. Then we have read all the 13 abstracts and selected ten articles. After reading the full texts finally eight articles were selected for the current review study.

#### Criteria for evaluating selected studies

The evaluation criteria of reference studies were based on the check-list developed by Drummond, et al.^14^ All the eight articles were then vividly reviewed and evaluated.

## RESULTS

Eight articles were finally evaluated in accordance with Drummond’s guidelines^14^. All of these papers found explaining the research question and the economic evaluation explicitly. The main and overall results are presented in detail in Table 1, and the parameters of cost-effectiveness analyses from these articles are discussed in table 2 and 3.

**Table 1:** Review of articles

Author & Date	Place of Study	Methods	Main Findings	Author’s Conclusions
Vermeer F, et al. 1988^15^	The Netherlands	A 12 month follow up of 269 patients allocated to thrombolytic treatment and of 264 allocated to conventional treatment.	Quality adjusted mean survival for inferior infarction patients was 307 days (out of 365days) vs 300 days in the thrombolysis & control group; in patients with anterior infarction, it was 38 days longer in the thrombolysis group.	The higher costs of patients receiving thrombolytic were mainly the result of acute catheterization.
		The costs of medical treatment, including medication, hospital stay, cardiac catheterisation, coronary angioplasty, and bypass surgery were considered.	Medical cost: after MI averaged Dfl 21000 for interior infarction & Dfl 20000 for anterior infarction in the control group; Dfl28000 & Dfl29000 in the thrombolysis group.	Thrombolytic increased life expectancy particularly in patients with anterior infarction (2.4 years).
		Mean survival was calculated and survival was adjusted for impaired quality of life.	Additional costs per year of life gained were Dfl 3800 in patients with anterior infarction and Dfl 10000 in inferior infarction.	
Levin LA, et al. 1992^16^	Sweden	Randomized double-blinded placebo-controlled study comparing rt-PA with placebo in patients with suspected AMI.	The direct costs were significantly higher among rt-PA group than the placebo group due to the cost of thrombolytic drug.	Cost-effectiveness of intravenous thrombolysis treatment in suspected MI is higher than other medical treatment of coronary heart disease.
		ASSET data and the specific economic data are applied.	The indirect costs are 4190 SEK lower in the rt-PA group as a larger proportion of rt-PA patients returned to work during the follow-up period.	
		Direct medical costs & indirect costs (productivity loss) were computed.	The rt-PA mean patient cost(total cost) is 5700 SEK higher than the placebo group. Rt-PA therapy inceses life expectancy by about 1.25 years with a 6% mortality rate and it increases as the mortality rate falls.	
		The health benefits are analyzed by the treatment effects on mortality and the effects of treatment on the patient’s quality of life which are measured with the NHP questionnaire.	The cost-utility ratio of rt-PA varies from 3260 SEK to 6310 SEK per QALY gained & the cost-effectiveness ratio varies from 3090 to 5970 SEK.	
		The life expectancy is estimated by the DEALE method.		
Machecourt J, et al, 1993^17^	France	Double-blind, doubledummy procedure. 180 patients were randomized in a 12-month period with either anistreplase or alteplase.	The cost ranged from 6570 ECU to 6050 ECU per patient, without any significant thrombolytic agent related difference: the total cost of the hospital phase was 6460 ECU for alteplase, 6570 ECU for anistreplase and 6050 ECU for streptokinase (NS). The cost/efficacy ratio was 548 ECU for alteplase, 570 ECU for anistreplase and 405 ECU for streptokinase.	No difference observed in efficacy between the three thrombolytic agents for the three left ventricular parameters and for the patency of the infarctrelated artery.
		Analysis of efficacy including secondary effects of treatments, cost assessment, and variance analysis were conducted	The cost for deceased patients was lower than for those who survived with the total costs of 4466 ECU vs 6512 ECU.	The ICER is similar for anistreplase, alteplase and streptokinase, with a slight advantage for streptokinase.
Rawles JM, 1997^18^	Scotland	Double-blind, randomized, placebo-controlled small trial.	Age, treatment delay and time of presentation determine the outcome at 30 months according to logistic regression.	The pre-hospital therapy of anistreplase is more effective than in-hospital use of streptokinase.
		“Home injection” & “hospital injection” was given. Multivariate analysis with logistic regression.	In the 5-year-follow up period, patients in the home groups live more than 6 months longer on average.	
		Comparative costeffectiveness analysis with Streplase from GUSTO trial.		
Franzosi MG, et al. 1998^19^	Italy	RCCT (2*2 factorial design)- patients were randomly assigned to receive oral lisinopril or open control &to receive nitroglycerin or open control	Costs for most of the concomitant treatments were $US67472 vs $US32677 (control group vs lisinopril group).	The comparative costeffectiveness ratio for the use of lisinopril was $US2080 per life saved.
			The comparative cost-effectiveness ratio for the use of lisinopril was $US2080 per life saved at 6 weeks post MI.	
		Only direct costs regarding publicly financed healthcare were considered	The cost-effectiveness ratios varies from $US1121 to $US40910 per life saved, after conducting the initial sensitivity analysis.	The cost-effectiveness ratios were more favorable in subgroups with higher absolute survival benefit.
		CEA is based on the crude rate of survivors from the 6-week treatment of AMI with Lisinopril (no discounting). Sensitivity analysis was performed.	After the second sensitivity analysis, the comparative cost-effectiveness ratio for the use of lisinopril increased to $US4530 per life saved.	
			The number of lives saved per 1000 patients treated with lisinoprilwas greater for older patients than for younger patients.	
Lorenzon R, et al. 1998^20^	Italy, UK Germany, USA	CEA of results from the 30-day GUSTO trial (no discounting of costs& benefits):	The cost for each extra life saved in Germany, Italy, U.S.A., is 31%, 45%, 97%, higher than that in the U.K.	Cost-effective analysis is an “affair of state”.
		The incremental costs for each life saved for the ageselective and site-selective protocols were considered.	Age-selective protocol: there would be 64 deaths & 9 patients saved per 1000 patients treated.	Age-selective use of rt-PA is inappropriate; siteselective use of tPA in anterior AMI halves the costs for each extra life saved.
		Sensitivity analysis on the results performed No discounting for costs and effects.	Cost for each of these extra lives saved would be $U.S 144,126, $U.S 159,883, $U.S 109,848, $U.S 216,142 in Germany, Italy, UK and the US respectively Site-selective protocol: 65 deaths/1000 patients treated&8 patients per 1000 patients treated saved, and the cost for each of these extra lives saved would be $U.S 71,858, $U.S 79,715, $U.S 54,769, $U.S 107,764 in Germany, Italy, UK and the US	
Marcoff L, et al. 2009^21^	France	ExTRACT-TIMI 25 – a large, randomized, multinational trial at 674 sites in 48 countries.	The net clinical benefit compared with UFH: relative risk reduction 17%, 95% CI:0.10-0.23, p< 0.001; absolute risk reduction 2.1%.	Enoxaparin is both effective and cost effective compared with UFH – also confirmed by probabilistic sensitivity analysis.
		QALY and mean cost compared between Enoxaparin & UFH group. discounted at 3% annually.	There is 90% probability of the enoxaparin being cost effectiveness at the $50,000 threshold.	
		Cost and effectiveness analysis were conducted for subgroups measured by NMB &NHB Probabilistic Sensitivity analysis of costs and LYG.		
Welsh RC et al. 2009^22^	Canada	Randomized, double-blind, double-dummy, parallel group, of 20,506 patients in at 674 sites in 48 countries, and 118 patients in Canada. Enoxaparin or matching placebo.	When considering the marginal time horizon and allowing clinical benefit to be accounted for, enoxaparin was found to be cost-effective with an ICER of $4,930 and 99% probability of the costeffectiveness ratio being less than $20,000.	Long-term clinical data are required to confirm the assumption that the difference in survival between arms of the study does not widen or close after 30 days.
		LYG was used as the outcome measure; societal perspective (5% discounting rate). Costs of treatment, ICER, LYL were computed.	A reduction of treatment duration reduced the ICER to 1,176/LYG.	
			A drop of 15% in marginal costs resulted in an ICER of $4,191;an increase of 15% resulted in an ICER of $5,670.	
		Sensitivity analysis performed.		

**Table 2:** Review of alternatives being compared, perspectives of study and source of data in selected articles

Author	Alternative being compared	Views of Point	Source of data
Vermeer F, et al. 1988^15^	Thrombolytic group and control group	Not Stated	Not Stated
Levin LA, et al, 1992^16^	The rt-PA group and the placebo-treated patients	Not Stated	The original ASSET data & the economic data were from the participating centers
Machecourt J, et al. 1993^17^	Comparison of the efficacy of streptokinase, alteplase and anistreplase; comparision of hospital costs of the there thrombolytic agents	Not Stated	From preliminary results of the ISIS III study
Rawles JM, 1997^18^	Thrombolytic treatment with anistreplase at home or in hospital later; Marginal cost per life-year with prehospital anistreplase versus streptokinase was stated.	Not Stated	Not Stated
Franzosi MG, et al. 1998^19^	The lisinopril group and the control group	Italian National Health Service Perspective	Cost data gathered from GISSI-3 study and the whole price of lisinopril in 1993 in Italy
Lorenzon R, et al. 1998^20^	Thrombolytic treatments with tPA and Streptokinase were compared	Not Stated	Efficacy data deprived from GUSTO trial; cost data on streptokinase & tPA from national formulary
Marcoff L, et al. 2009^21^	Cost and cost-effectiveness of interventions with enoxaparin and UFH	Societal Perspective	Patient-level data were used directly from the ExTRACT-TIMI 25 trial
Welsh RC et al. 2009^22^	Cost and cost-effectiveness of interventions with enoxaparin and UFH	Societal Perspective	CEA used data from the Ex-TRACT-TIMI 25 trial

**Table 3:** Evaluation of the articles according to Drummond’s parameters

Author	Marginal Analysis	Statistical Analysis	Sensitivity Analysis	ICER		Scope of Study	
					Cost	Outcome	Accrued over time
Vermeer F, et al. 1988^15^	Not Stated	Stated	Not Stated	Not Stated	Yes	Yes	No Discounting Stated
Levin LA, et al. 1992^16^	Not Stated	Stated	Not Stated	Stated (Costeffectiveness and cost-utility ratios)	Yes	Yes (QALY as cost-utility measure)	Discounting Stated 5%
Machecourt J, et al. 1993^17^	Not Stated	Stated	Stated	Stated	Yes	Yes	No Discounting Stated
Rawles JM, 1997^18^	Stated (Marginal costs	Not Stated	Stated	Not Stated	Yes	Yes	No Discounting Stated
Franzosi MG, et al. 1998^19^	Not Stated	Stated	Stated	Stated (Incremental costs for each extra life saved)	Yes	Yes	No Discounting Stated
Lorenzon R, et al. 1998^20^	Not Stated	Stated	Stated	Stated (Cost per additional survivor)	Yes	Yes	No Discounting Stated
Marcoff L, et al. 2009^21^	Not Stated	Stated	Stated	Stated (measured by LYG, QALY)	Yes	Yes	Discounting Stated 3%
Welsh RC et al. 2009^22^	Not Stated	Stated	Stated	Stated (measured by LYG)	Yes	Yes	Discounting Stated 5%

The review highlights that effectiveness of MI treatment depends on some pre-defined conditions (Table 1). Vermer et al^15^ indicated that the benefits of thrombolytic treatment were most prominent in patients with anterior infarction as it increases life expectancy. The Swedish study by Levin et al. found that the cost-effectiveness of intravenous thrombolysis treatment in suspected MI is higher than that in other medical therapies of coronary heart disease.^16^ Machecourt et al. presented that streptokinase, anistreplase and alteplase are of similar efficacy.^17^ Rawles illustrated that the prehospital uitlization of anistreplase could increase the immediate survivals of MI accidents and survivals in longer period of 5 years.^18^ Franzosi et al. came to conclusion that isinopril is very beneficial for MI patients with high absolute survival benefit.^19^ Lorenzoni et al. stated that selective use of Streptokinase in combination of tPA is more effective than the exclusive use of rt-PA.^20^ A recent study from France by Marcoff et al. demonstrated that Women appear to have similar relative and greater absolute risk reductions compared to men when enoxaparin is used with lytic therapy. Total lifetime costs (30-day costs plus the costs beyond trial period) were $1,207 higher in the Enoxaparin group due to the longer life expectancy of patients in Enoxaparin group.^21^ An ICER of $4,930 per life years gained was very favorable compared with unfractioned heparin in the base case study from Welsh et al.^22^

The papers by Vermeer et al.^15^ Franzosi et al.^16^ and Levin et al.^17^ do not make any comparisons between different treatments. The study by Vermeer et al.^15^ analyzes the cost-benefit of early treatment with intracoronary streptokinase and found streptokinase cost effective in treatment in patients with extensive anteroseptal infarction. Franzosi et al^16^ study the cost-effectiveness analysis of the early use of Lisinopril (ACEI) which is more favorable compared with other cost-effectiveness analyses. The paper by Levin et al.^17^ examines the costs and effects of intravenous thrombolytic therapy of rt-PA and shows it cost-effective in comparison with other coronary heart disease treatments. The studies by Lorenzoni et al.^18^ and Machecourt et al.^19^ compare the different competing thrombolytic agents. Lorenzoni et al.^18^ find that the cost-efficacy of rt-PA and streptokinase differs among countries attributing to the costs difference and prove that the selective use of rt-PA and streptokinase is more cost-effective than the exclusive use of rt-PA. The study by Machecourt et al.^19^ demonstrates that there is no significant difference in efficacy between anistreplase, alteplase and streptokinase and the costs of them have little effect on the total cost of MI. The study by Rawles.^20^ reveals that prehospital use of Anistreplase increases the survival of patients than the hospital administration of Anistreplase. It shows that prehospital therapy with Anistreplase is highly cost effective compared with streptokinase given in hospital, and the marginal cost-effectiveness ratio is much lower than that for TPA versus streptokinase derived from GUSTO trial. Marcoff et al.^21^ explains that Enoxaparin is cost effective when replaced with UFH as adjunctive therapy for fibrinolysis for patients with STEMI in both short- and long-term. Welsh et al. also compares these two drugs, and concludes that Enoxaparin is cost-effective with a modest increase in direct medication costs.^22^

Perspectives are not specified in the studies by Vermeer et al.,^15^ Lorenzoni et al.,^18^ Rawles,^20^ Machecourt et al.^19^ and Levin et al.^7^ Study by Franzosi et al.^16^ applies a perspective of Italian National Health Service while studies from Marcoff et al.^21^ and Welsh et al.^22^ are of societal perspective. All of these studies state the data sources but the two studies by Rawles^20^ and Vermeer et al.^15^ ICER is not analyzed in these studies whereas others do (Table 2).

Levin et al.^7^ do not state sensitivity analysis while the rest of studies performed this analysis. In all the studies, costs are interpreted in terms of drug prices, hospitalization, salaries of medical staff, etc. ICER is computed except in the studies by Rawles^20^ and Vermeer et al.^15^ LYG and QALY is used as outcome measurements by Marcoff et al.,^21^ Levin et al,^16^ and Welsh et al.^22^ Quality adjusted mean survival and life expectancy is used to assess health outcome in the study by Vermeer et al.^15^ Costs for each extra life saved are used in other cost-effectiveness analyses. Levin et al.^17^ and Welsh et al.^22^ utilize 5% discount rate and Marcoff et al^21^ used 3% discount rate, but costs and effects are not discounted in other studies (Table 3).

## DISCUSSION

Among the 8 articles selected, five studies focus on thrombolytic, and the other three studies are about ACEI and heparin. All of these papers are in line with the objectives of this study. Most studies were implemented in developed world and the ExTRACT–TIMI 25 study^10^ was conducted in 48 countries, including both developed and developing countries. ICER was assessed in six out of the eight articles to compare the costs and health effects between alternative medications. ICER was expressed in different effect units.

In the process of literature review, it was found that economic evaluation studies of thrombolytic treatment of MI started from late 1980s^16^ and there were more studies on economic evaluations of medication (thrombolytic and other pharmaceuticals) therapy of MI in the 1990s.^16^–^20^ In addition, more studies on surgical treatments in the last decade due to the magnificent achievements in medical technologies. Besides, similar studies tend to be carried out in developing world attributing to a higher prevalence of MI, and the ExTRACT-TIMI25 Trial^9^–^10^ is one example.

It was found that five of the reference studies researched on thrombolytic^15^,^16^–^20^ and six studies calculated incremental cost-effectiveness ratio (ICER). Vermeer et al.^15^ only studied the costs and outcome for the cost-benefit analysis of streptokinase and Rawles^18^ utilized marginal analysis to measure the cost-effectiveness of Anistreplase. Among the other three thrombolytic studies, ICERs were evaluated. Lorezoni et al.^20^ reported the values of ICERS of rt-PA and streptokinase which vary greatly among four countries because of difference in the costs of drugs; and the site-selective use of both rt-PA and streptokinase halves the cost-effectiveness ratio compared to the exclusive use of rt-PA for AMI. Machecourt et al.,^17^ proved that there is no significant difference between anistreplase, alteplase and streptokinase in cost/efficacy ratio, and this finding is different from a previous study^23^ as the previous research did not incorporate costs in the hospital phase. Machecourt et al^17^ also illustrated that streptokinase is consistently of slight advantage when compared with the other two thrombolytic agents. Levin et al.^16^ concluded that intravenous rt-PA is cost-effective by comparing the ICER with that of other treatments of coronary heart diseases, taking the study by Vermeer et al.^15^ for example. The comparison in the former study was made among three thrombolytic agents, whereas the latter study compared intracoronary streptokinase with beta adrenergic antagonists and coronary artery bypass surgery. The difference between the studies by Machecourt et al.^19^ and Levin et al1^7^ is assumed to be caused by a range of reasons, e.g. different comparison methods, prices of drugs, costs items included, different values of the currencies in France and Sweden.

In studies regarding ACEI^19^ and enoxaparin and UFH^21^,^22^, all of them included ICERs. Franzosi et al.^19^ found that the ICER of lisinopril is lower compared with that of other therapies for AMI, given similar benefit and routinely utilization. E.g. the ICER of lisinopril was $US 2080 per life year saved compared with $US 32687 per life year saved of alteplase (rt-PA) which was found to be more cost-effective in the study by Levin et al.^16^ However, deviation might occur when comparing different cost-effectiveness analyses in different settings, due to a difference in health care systems, drug prices, cultures, etc. On the contrary, when it comes to enoxaparin and UFH, both Marcoff et al.^21^ and Welsh et al.^22^ reached the same conclusion that using enoxaparin instead of UFH in conjunction with fibrinolysis in STEMI patients is cost effective by evaluating the ICERs, and the results were found to be in line with other studies.^24^,^25^

All the studies that we analyzed, excluding the multiple-country trial ExTRACT-TIMI 25,^10^ reported achievements in many developed countries in the research of the health care expenditures, health outcome and effects of various health programs for treatments of MI. However, very few researches were done in the less developed countries and it is very difficult for patients with MI to get access to the medical service. The inequality is assumed to be resulted from inappropriate distribution of healthcare resources, lower income, high costs of treatments for MI, underdevelopment in transportation in developing world.

The present study also found that treatment strategies such as PCI, stenting angioplasty, CABG are more commonly used in recent decades than that in the past. Meanwhile, medications still play an important role. For instance, two American specialists of cardiology points out that if PCI capability is not available or will cause a delay over 90minutes, then the optimal approach is to administer thrombolytics within 12 hours of onset of symptoms in patients with ST-segment elevation greater than 0.1 mV or more continuous ECG leads, new left bundle-branch block or anterior ST-depression consistent with posterior infarction.^26^ Streptokinase, APSAC or anistreplase and rt-PA, which are commonly used, are found to have no significant difference in efficacy rates with respect to hospital mortality and imminent risk of massive haemorrhage.^7^ The benefit of thrombolytic agents is dependent on the early administration of the drug, and Rawles^20^ illustrated the timely implementation of anistreplase. Despite the substantial overall benefits of the medical approaches, adverse effects and limitations of the pharmaceuticals must be taken into account. E.g. antitrombotic agent enoxaparin has been found to lead to an increase in major bleeding in patients with STEMI;^27^ rt-PA show a significant efficacy in patients younger than 75 years and in patients with anterior acute myocardial infarction, but no evident advantage in those over 75 and with non-anterior acute myocardial infarction.^28^ Hence, to make most of the medications of MI, subgroup analysis is required.

Myocardial infarction is a major cause of morbidity and mortality throughout the world. MI treatment is one of the most expensive interventions for acute coronary syndromes, exerting a detrimental influence on the economic growth and well being. The burden of disease falls both in developed and developing countries. Thus, it is of great importance to gain deeper insights to MI, and seek for appropriate treatments which are cost effective to the whole population.

Economic evaluation of MI treatments provides a great deal of information to decision makers to identify and opt between different regimens. It is vital to state the perspective of a study, as a program appears unattractive and costly from one viewpoint may be considered effectively applicable from other viewpoints^14^. Yet, they were not specified in most of the studies above. None of these studies except the one by Rawles^20^ did marginal analysis and Levin et al.^17^ did not perform sensitivity analysis, though both analyses are important in economic evaluations. ICER, which is crucial in economic analysis, is reported explicitly in all the studies but the ones by Vermeer et al.^15^ and Rawles.^20^ It is recommended that future cost-effectiveness studies should include all the important aspects of an economic evaluation in particular ICER.

This study found that in many countries various medications including thrombolytic agents, ACEI and heparin are cost-effective when administered for MI treatment. It is also found that five of eight studies focus on thrombolytic therapies. The prompt delivery of thrombolytic therapy increases the efficacy which will result in a decrement on the costs of further treatment. To sum up, it implies that thrombolytic is very cost effective for MI in a range of countries with different settings.

It is also suggested that similar studies be undertaken in developing countries where there is a high burden of disease but lack of relevant interventions and studies.

### Abbreviations

CEA = Cost-effectiveness Analysis; AMI = Acute Myocardial Infarction; tPA = Tissue Plasminogen Activator; RCCT = Randomized controlled clinical trial; RCT = Randomized controlled trial; NHP = Nottingham Health Profile; rt-PA = Recombinant tissue plasminogen activator; LMWH = Low-molecular weight heparin; UFH = unfractionated heparin; LYG = life-years gained; QALY = quality-adjusted life-year; STEMI = ST-segment elevation myocardial infarction; ExTRACT-TIMI = Enoxaparin and Thrombolysis Reperfusion for Acute Myocardial Infarction Treatment Thrombolysis in Myocardial Infarction 25; ICER = Incremental Cost-effectiveness Ratio; PCI = Percutaneous coronary intervention; CABG = Coronary artery bypass grafting; ACEI = Angiotensin-converting enzyme inhibitors; APSAC = Anisoylated plasminogen streptokinase activator complex; GISSI-3 = Gruppo Italiano per lo Studio della Sopravvivenzanell’ Inarto; ISIS-3 = Third International Study of Infarct Survival; ASSET = Anglo-Scandinavian Study of Early Thrombolysis; GUSTO = Global Utilization of Streptokinase and Tissue Plasminogen Activator for Occluded Coronary Artery
